# Bilateral Intertrochanteric Femur Fractures in a Paraplegic Patient: A Case Report

**DOI:** 10.7759/cureus.54883

**Published:** 2024-02-25

**Authors:** Anthony Forrest, Samuel G Eaddy, Zachary W Fulton, Benjamin Boothby

**Affiliations:** 1 Orthopedic Surgery, Mercy Health St. Vincent Medical Center, Toledo, USA

**Keywords:** spinal cord injury, cephalomedullary nail (cmn) fixation, parapalegic, it femur fractures, bilateral intertrochanteric femur fractures

## Abstract

Bilateral intertrochanteric (IT) femur fractures are rare, and appropriate evaluation and treatment can vary depending on concurrent patient comorbidities. Even less has been described for patients with bilateral IT fractures with pre-existing paraplegia. This case report describes the unique case of a 72-year-old paraplegic female who presented with bilateral IT femur fractures due to a wheelchair accident. The patient was treated with single-stage bilateral cephalomedullary nail fixation so she could effectively transfer to and from the wheelchair with less pain and a greater chance of fracture union. At the last follow-up, the patient’s pain had resolved and she was able to transfer as effectively and safely as her pre-injury baseline. Single-stage cephalomedullary fixation of bilateral IT femur fractures is indicated in the paraplegic population to relieve pain and improve effective safe transfers for daily activities.

## Introduction

Hip fractures are a common injury, with more than 300,000 occurring in the United States each year [1]. Intertrochanteric (IT) hip fractures account for 42% of all hip fractures [2], and are typically unilateral, in females older than 65 years of age, and due to a ground level fall in the setting of osteoporosis. Bilateral IT fractures are rare, with an estimated incidence of less than 0.3% in the general population [3]. The mechanism of injury for bilateral IT fractures typically involves high-energy trauma or an inciting event such as a seizure or an electrocution [4]. Moreover, bilateral IT fractures may also present following low-energy mechanisms in patients with underlying locally destructive disease processes such as multiple myeloma and metastatic disease, or concomitant factors like osteoporosis, chronic renal failure, chronic steroid use, electroconvulsive therapy, long-term bisphosphonate use, and certain metabolic disorders [5-8].

The bone-weakening effects of paralysis from spinal cord injury have also been described, where the bone mass of the epiphyses and diaphysis exponentially decreases post-injury until reaching a new steady state after 3-8 years [9]. As a result, these patients are at an increased risk for pathologic fragility fractures [10-12]. A comprehensive literature search yielded no results describing the unique occurrence of bilateral IT fractures in paraplegic patients. With informed patient consent, we present a case report of bilateral IT femur fracture fixation in a paraplegic woman with short-term follow-up.

## Case presentation

A 71-year-old female presented to the emergency department of a level 1 trauma center following a fall from a wheelchair-accessible van. At the time, the patient was secured to her motorized wheelchair by a waist belt and fell directly on her back. She did not hit her head or experience loss of consciousness.

The patient has an 11-year history of a permanent incomplete spinal cord injury at the level of C6/7 (Grade B American Spinal Injury Association (ASIA) Impairment Scale). Other pertinent medical history includes a stage IV sacral decubitus ulcer, prior methicillin-resistant *Staphylococcus aureus* (MRSA) infections and a recent urinary tract infection associated with an indwelling urinary catheter which was treated with a 14-day course of meropenem. On examination, the patient had no gross motor function in the bilateral lower extremities and appeared thin with disuse. She had pain with palpation and log roll of her bilateral hips.

**Figure 1:**
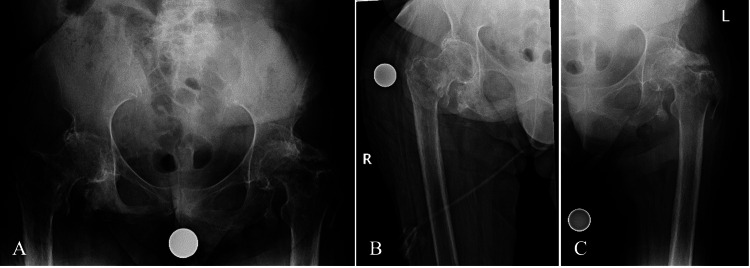
Initial injury radiographs A) Anteroposterior view of the pelvis demonstrating a left intertrochanteric (AO: 31A2.2) and right intertrochanteric (AO: 31A2.1) femur fracture. Anteroposterior view of B) the right hip and C) the left hip further demonstrates the fracture patterns. AO: Arbeitsgemeinschaft für Osteosynthesefragen

Initial plain radiographs demonstrated bilateral displaced IT femur fractures (Arbeitsgemeinschaft für Osteosynthesefragen/Orthopedic Trauma Association (AO/OTA) type 31A2.1 (right) and 31A2.2 (left)) and a previously healed left femoral neck fracture (Figure 1). It was discussed that given her paraplegic state, nonoperative treatment would be a reasonable option. With a short trial of nonoperative treatment during her hospital stay, she experienced pain in her hips when repositioning and transferring from her wheelchair. Transferring was unsafe, painful, and limited due to her clinical state. Thus, she ultimately elected to proceed with operative intervention to stabilize the fractures and allow a greater chance of union and safe mobilization while transferring for daily activities. Cefepime was recommended due to her history of a recent urinary tract infection, prior MRSA infections, and stage IV sacral decubitus ulcer. Her pre-operative hemoglobin was 12.2 g/dL, and she was cleared by her ancillary medical teams for orthopedic surgical intervention.

**Figure 2:**
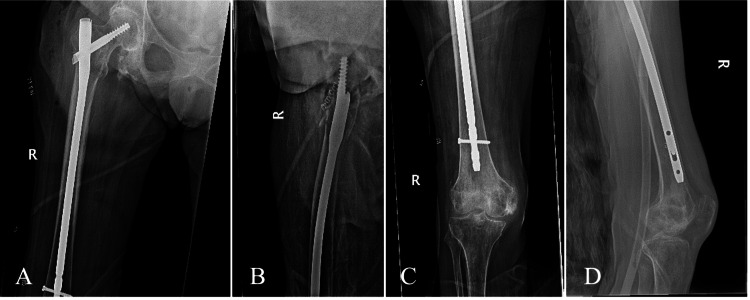
Immediate postoperative radiographs of the right femur demonstrating fixation of the right IT femur fracture with an unreamed intramedullary long nail A) Anteroposterior view of the right proximal femur. B) Lateral view of the right proximal femur. C) Anteroposterior view of the right distal femur. D) Lateral view of the right distal femur. IT: Intertrochanteric

**Figure 3:**
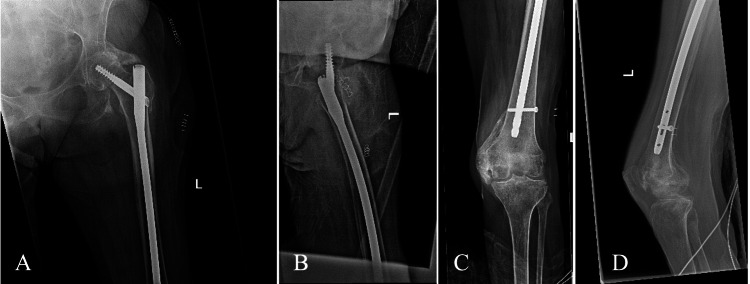
Immediate postoperative radiographs of the left femur demonstrating fixation of the left IT femur fracture with an unreamed intramedullary long nail A) Anteroposterior view of the left proximal femur. B) Lateral view of the left proximal femur. C) Anteroposterior view of the left distal femur. D) Lateral view of the left distal femur. IT: Intertrochanteric

Surgical consent was obtained pre-operatively. Surgery was performed under general anesthesia with the patient positioned supine on a Hana fracture table in the scissor leg position. Closed reduction of the left IT fracture was performed with axial traction, and internal and external rotation, until acceptable near anatomic reduction was obtained. The fracture was then fixed with an unreamed intramedullary long nail (Trochanteric Fixation Nail-Advanced (TFNA), 10 x 380 mm, 125°, DePuy Synthes, Paoli, United States) with a single distal interlocking screw. After repositioning the legs, the right hip was then fixed in a similar fashion with an unreamed intramedullary long nail (TFNA, 10 x 380 mm, 125°) and a single distal interlocking screw. Immediate postoperative radiographs indicated appropriate fracture reduction, fixation, and without acute complications (Figures 2, 3). She was given no postoperative restrictions following her surgery. There were no intraoperative complications.

In the post-anesthesia care unit, the patient became hypotensive and tachycardic and the decision was made to transfer her to the intensive care unit (ICU) for further monitoring. An electroencephalogram demonstrated moderate to severe encephalopathy, and mounting concern for drug-induced neurotoxicity, which led to her antibiotics being switched from cefepime to Zosyn. Her mentation improved significantly, and she was subsequently transferred to inpatient monitoring after three days in the ICU. The infectious disease then changed zosyn to a seven-day course of IV meropenem for better UTI coverage. On postoperative day eight, the patient was discharged to a skilled nursing facility.

**Figure 4:**
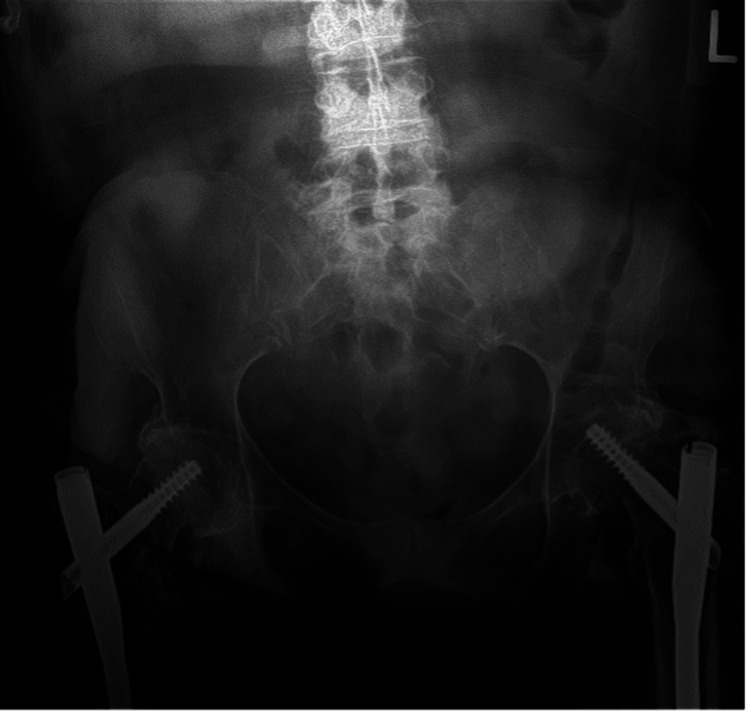
Six-week postoperative anteroposterior radiograph of the pelvis demonstrating mild collapse of the left proximal fracture and subsequent 1 mm of screw cut-out

**Figure 5:**
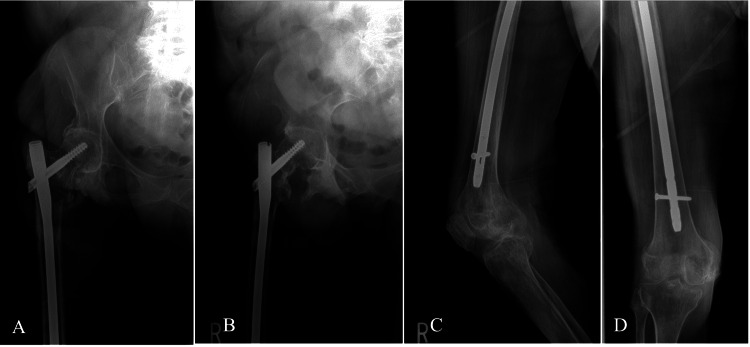
Six-week postoperative radiographs of the right femur A) Anteroposterior view of the right proximal femur. B) Lateral view of the right proximal femur. C) Anteroposterior view of the right distal femur. D) Lateral view of the right distal femur.

**Figure 6:**
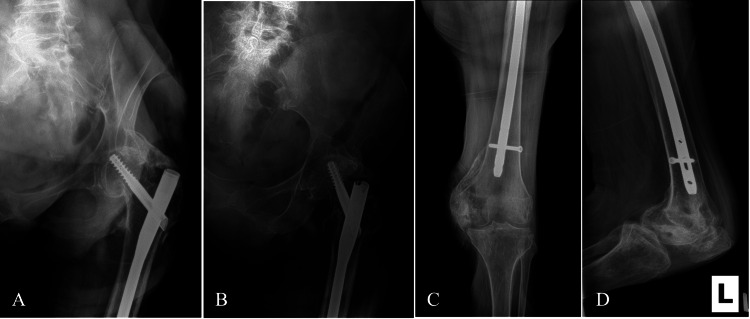
Six-week postoperative radiographs of the left femur A) Anteroposterior view of the left proximal femur demonstrating mild collapse of the fracture and 1 mm of screw cut-out. B) Lateral view of the left proximal femur. C) Anteroposterior view of the left distal femur. D) Lateral view of the left distal femur.

Two weeks postoperatively, she had a mild reduction in pain and no wound complications. At the patient’s six-week follow-up, her bilateral hip pain was further reduced to a near pre-injury state. Radiographs were obtained at this visit, demonstrating intact hardware (Figures 4-6). There were radiographic signs of screw cut-out within the left femoral head, likely due to the prior femoral neck fracture that was presumed healed at the time of surgical intervention. However, her pain was well-controlled without narcotic medication, and her transfer capabilities were back to baseline. At the patient’s 12-week postoperative tele-visit, she expressed that she was doing well and had gotten back to her pre-injury level of activity without pain. Unfortunately, six months postoperative the patient passed away in hospice care due to unrelated causes.

## Discussion

While IT femur fractures are common, their bilateral occurrence is rare. Bilateral IT fractures most often arise from high-energy trauma, though this fracture pattern can also be predisposed in patients with a varying degree of superimposed comorbidities. Moreover, spinal cord injuries resulting in paralysis have a well-established detrimental effect on lower extremity bone density and their management requires some unique considerations [13-17].

A review of the literature was performed to identify all reported cases of bilateral IT fractures, and to the best of our knowledge, this is the first report describing this unique fracture pattern in a paraplegic patient. Galan-Olleros et al. [18] evaluated 25 patients with bilateral extracapsular hip fractures or combined intra-extracapsular fractures in the literature; of those cases, only 12 were elderly patients and none were paraplegic.

In this patient, there are various documented reasons to justify operative and nonoperative intervention as well as single versus staged procedures, intramedullary versus extramedullary fixation, short versus long nails, and reamed versus unreamed medullary canals [14,15]. Nonoperative management techniques are essentially obsolete in the 21st century and can contribute to malunion, leg length discrepancy, joint contractures, and complications resulting in death [13,15].

In this patient we opted for bilateral long cephalomedullary nail fixation to stabilize the proximal fracture and protect the entire femur, thereby limiting the risk of potential peri-implant fracture. We performed an unreamed nailing technique bilaterally to shorten total operative time and to reduce the theoretical risk of reaming-induced embolization. Prophylactic anticoagulation with Lovenox 30 mg twice daily was also initiated on postoperative day one. Furthermore, a decision was made to perform both surgeries during one anesthetic event to minimize time and complications from general anesthesia.

This case report is limited by the patient’s short follow-up course. Without extended postoperative follow-up, we are unable to definitively state the long-term outcome of her treatment. However, her clinical progress at 6 and 12 weeks postoperative was reassuring. Likely, the radiographic findings of the left hip screw cut-out are multifactorial: poor bone quality would certainly contribute, and if the previous femoral neck fracture was not entirely healed this would increase the risk of collapse with concomitant screw cut-out. When the tip-apex distance is greater than 25 mm, there is an increased risk of screw cut-out [19]. However, the tip-apex distance in our patient during the immediate postoperative period was measured to be 14 mm, which would indicate that patient factors such as poor bone quality or prior unhealed femoral neck fracture contributed to screw cut-out rather than intra-operative measures. Given the patient’s disuse osteopenia, utilization of a cement-augmented cephalomedullary screw with injectable polymethylmethacrylate would have been a good addition to enhance proximal fixation and prevent screw cut-out.

Cochran et al. [13] originally reported that surgical intervention was more likely to occur in paraplegic patients with lower extremity fractures who were higher performers at baseline, such as wheelchair athletes. Sugi et al. [16] found in their cohort that all wheelchair-bound patients treated with open fixation had much shorter hospital stays and earlier mobilization for rehabilitation. Those patients also went on to successful bony union, reported significantly improved pain scores, and achieved good functional outcomes overall [16].

## Conclusions

Few reports of bilateral IT fractures can be found in the literature, and this is the first known report of a paraplegic patient. Long-standing spinal cord injury may be an underappreciated risk factor for bilateral hip fractures, and these patients have unique surgical considerations. Bilateral surgical fixation appears to be an appropriate choice of treatment, especially if patients experience pain and instability when transferring. The decision to pursue surgical management, however, should consider the extensive risk factors that can accompany a patient with paraplegia.
